# Wearable Sensor-Based Prediction Model of Timed up and Go Test in Older Adults

**DOI:** 10.3390/s21206831

**Published:** 2021-10-14

**Authors:** Jungyeon Choi, Sheridan M. Parker, Brian A. Knarr, Yeongjin Gwon, Jong-Hoon Youn

**Affiliations:** 1 College of Information Science and Technology, University of Nebraska at Omaha, Omaha, NE 68182, USA; jyoun@unomaha.edu; 2 Department of Biomechanics, University of Nebraska at Omaha, Omaha, NE 68182, USA; sheridanparker@unomaha.edu (S.M.P.); bknarr@unomaha.edu (B.A.K.); 3 Department of Biostatistics, University of Nebraska Medical Center, Omaha, NE 68198, USA; yeongjin.gwon@unmc.edu

**Keywords:** accelerometer, wearable sensor, elastic net, ridge regression, timed up and go (TUG), gait analysis

## Abstract

The Timed Up and Go (TUG) test has been frequently used to assess the risk of falls in older adults because it is an easy, fast, and simple method of examining functional mobility and balance without special equipment. The purpose of this study is to develop a model that predicts the TUG test using three-dimensional acceleration data collected from wearable sensors during normal walking. We recruited 37 older adults for an outdoor walking task, and seven inertial measurement unit (IMU)-based sensors were attached to each participant. The elastic net and ridge regression methods were used to reduce gait feature sets and build a predictive model. The proposed predictive model reliably estimated the participants’ TUG scores with a small margin of prediction errors. Although the prediction accuracies with two foot-sensors were slightly better than those of other configurations (e.g., MAPE: foot (0.865 s) > foot and pelvis (0.918 s) > pelvis (0.921 s)), we recommend the use of a single IMU sensor at the pelvis since it would provide wearing comfort while avoiding the disturbance of daily activities. The proposed predictive model can enable clinicians to assess older adults’ fall risks remotely through the evaluation of the TUG score during their daily walking.

## 1. Introduction

The older adult population worldwide is rapidly increasing due to the advancements in medical science. Subsequently, health care issues for this population group are also emerging [1]. The aging of the human body has been shown to reduce one’s balance, resulting in a decline in walking function, and is highly related to the incidence of injury in the elderly population [2]. In particular, falls account for a considerable number of injuries to the elderly population [3]. Walking is the most basic movement in daily life, a crucial element of quality of life, and a good indicator of health [4]. Many gait analysis studies have been conducted to identify and prevent the risk of falls for older adults [5,6,7]. Furthermore, common clinical measures, such as the Timed Up and Go (TUG) test, have been frequently used to assess not only the performance of walking and balance [8,9,10,11,12] but also the risk of falling [13,14,15,16,17,18] in older people since it is an easy, fast, and simple method of examining functional mobility and balance without special equipment. Typically, the clinical measures are performed under expensive laboratory settings that are only available in large urban areas. Therefore, a novel monitoring device is needed to monitor older adults’ mobility and predict their risk of falls using the TUG test in order to evaluate their health status.

With the advancement of wireless sensor technologies in the past few years, wearable sensors have been widely used to analyze human walking performance. These sensors offer a lot of practical benefits such as cost-effectiveness, convenience, and potential to provide health-related information [19]. This is particularly useful when interpreting gait characteristics and patterns to obtain a clear assessment of the associated health status. Buisseret et al. implemented an artificial intelligence algorithm to assess the risk of falls based on an autonomous wearable system [13]. Patel et al. found that the inertial sensors had a strong correlation with clinical fall risk tests in older adults [14]. Several studies also aimed to identify the relationship between the TUG test and the risk of falls in fallers or patient groups [15,16,17,18].

In this study, we constructed a model to predict the TUG test based on the ground walking data from wearable sensors in older adults in order to answer the following main research question:

- “Can the TUG test be predicted by normal ground walking data?”

Several studies [20,21,22] identified the different stages of the TUG test using the actual TUG test or similar settings, but we aimed to estimate the TUG test using normal ground walking with daily-life settings. Saporito et al. introduced a remote mobility monitoring method by estimating TUG from free-living activities [23]. We compared our TUG prediction results with Saporito’s TUG estimation.

Furthermore, we investigated which sensor location was better for predicting TUG based on walking data to address another research question:

- “Which is the optimal sensor location between foot and pelvis placements to collect data for predicting TUG while walking?”

In many studies, various sensor locations were used for health and mobility assessment [5,12,13,14,15,19,20,21,22,23,24]. Obviously, a wrist type of sensor is less obtrusive and more user convenient. However, we did not only look for the most comfortable body location for sensor attachment but an optimal sensor location that provides us with the useful information to predict the TUG test. Specifically, for our task of TUG prediction, a foot or a pelvis sensor provides more accurate information on accelerations compared to other locations.

This study will help us identify and assess the health status based on relationships between wearable gait data and clinical testing data. Moreover, this could be used to expand our research to help clinicians make rational decisions by providing adequate information about the recovery process of older patients.

## 2. Materials and Methods

**Figure 1:**
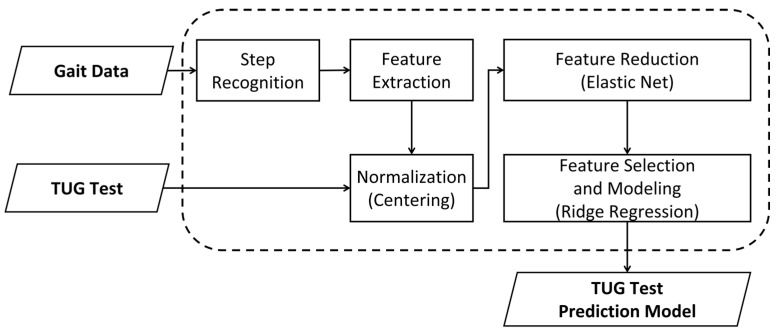
Framework for developing the timed up and go (TUG) test prediction model.

The framework in Figure 1 outlines the entire process of this study including data collection, data processing steps, and prediction model development.

### 2.1. Data Collection

#### 2.1.1. Participants

Data sets of 37 participants were used for the study analysis. The descriptive characteristics of the participants are shown in Table 1. The participant sample was pulled from two larger studies that incorporated the same outdoor walking task. The general inclusion criterion was being between the ages of 60 and 85 years. Exclusion criteria included a diagnosis of a neurological disorder (including stroke, traumatic brain injury, Alzheimer’s, and dementia), a diagnosis of osteoporosis, having underwent a total knee arthroplasty surgery and/or revision surgery, planning a staged total knee arthroplasty at the time of data collection, self-reported maximal joint pain of >3/10 (of the hip, knee, or foot), and/or doctor-diagnosed hip or knee osteoarthritis. All participants signed an informed consent approved by the University of Nebraska Medical Center Institutional Review Board (IRB 242-18-EP and IRB 654-16-EP).

#### 2.1.2. Walking Task

**Figure 2:**
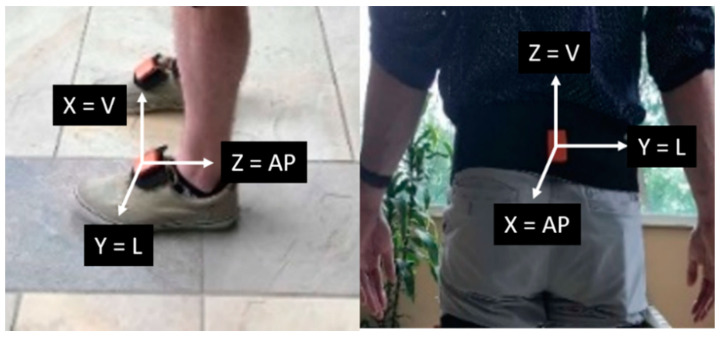
Sensor orientation on both the feet and pelvis. The sensor’s cartesian frame matches the walking direction (L = Lateral, V = Vertical, AP = Anterior-posterior).

Lower extremity accelerations were captured using a wireless inertial measurement unit (IMU)-based motion capture system (XSENS Awidna, Xsens Technologies B.V., Enschede, Netherlands). The IMU-based sensors were attached to the posterior pelvis and bilaterally to the foot segment using elastic Velcro straps as shown in Figure 2. The sampling frequencies of the IMU-based motion capture system were set to 60 Hz. The walking task was conducted in a neighboring community park across the street from the Biomechanics Research Building on the University of Nebraska Omaha campus. Participants were verbally instructed to walk at a comfortable pace following a circular sidewalk route. Three minutes of walking data were used for analysis. The outdoor walking condition was performed when outdoor temperatures were above 32 °F and at a minimum of 24 h after precipitation. The paved sidewalk was also cleared of obstacles prior to data collection.

#### 2.1.3. Functional Task

The TUG test is performed as a common clinical measure of evaluating fall risks in older adults [13,14,15,16,17,18]. Participants were instructed to start in a seated position on a standard-height chair, stand without using their arms or hands, walk 3 m, and return to a seated position. The time to complete the TUG test from rising from the seated position to chair contact was recorded using a standard stopwatch. The best time of two trials was used for data analysis.

### 2.2. Step Recognition

**Figure 3:**
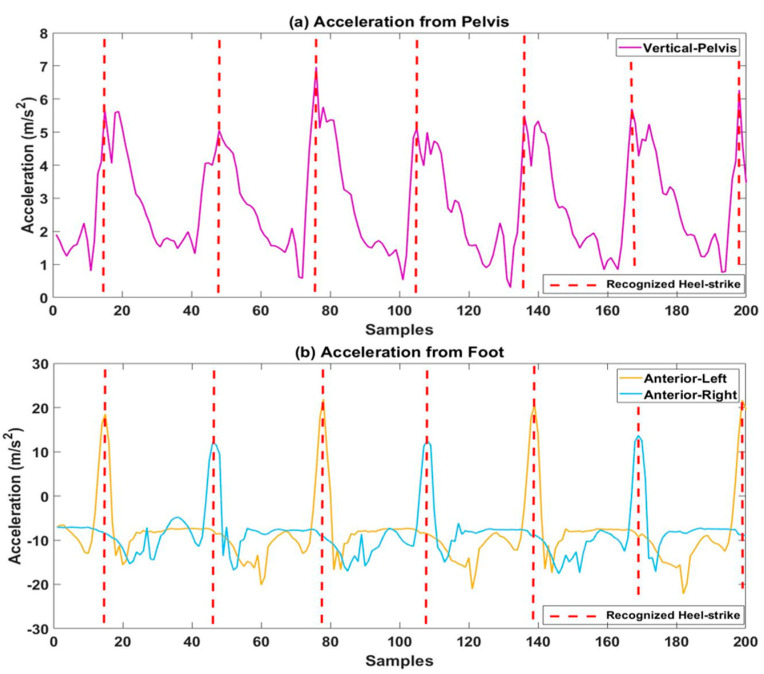
Recognized steps using accelerations from (**a**) pelvis and (**b**) foot. The step was recognized by detecting a heel-strike to another heel-strike. Red dotted lines represented the heel-strikes.

To recognize a step event, we used a peak detection method that detects the maximum peak of acceleration data. Based on our previous study [19], the peak detection method using two thresholds, a minimum-peak height and a minimum distance, successfully detects each step event with 100% accuracy. The minimum-peak height was set by computing the mean value of vertical and anterior-posterior accelerations for the pelvis and the foot, respectively, which was then used to find a peak larger than the minimum-peak height. The minimum-peak height was automatically determined for each subject. The minimum distance for all subjects was set by the number of samples that is 0.4 s at 60 Hz, as the average step time of a healthy person is 0.4 to 0.6 s [19]. Thus, the minimum distances for the pelvis and foot were 24 and 48, respectively. Since each foot sensor can only detect a stride event, the minimum distance for the foot was doubled. The minimum distance was also used to find the peak where the distance between the two peaks was longer than the minimum distance. For the foot sensor, we utilized an anterior–posterior (AP) acceleration to detect a heel-strike because the AP directional motion from the lower limbs prevails over the other dimensions [25]. A vertical acceleration was used for the pelvis sensor, as it showed more discernable pattern changes and the step-peaks were consistently captured similar to those from the foot sensor. A step was recognized by detecting a heel strike to another heel strike. On average, 369 (±27.6) steps were recognized and used for data analysis. Figure 3 shows recognized steps from both locations.

### 2.3. Feature Extraction and Normalization

A set of features was extracted from the raw acceleration data based on the recognized step events. For the two-foot sensors, once the step recognition was completed from each foot, we combined the detected peaks from the two sensors and raw acceleration data together to obtain the step-based features. In this study, we first extracted several features (i.e., M, M10, LM, VM, AM, LHM, LHS, VHM, VHS, AHM, AHS, and ST) that were used in our previous study [25], which have been helpful in predicting biomechanics in total knee arthroplasty patients. We also extracted additional features (i.e., MD, LMD, VMD, AMD, M30, LM30, VM30, and AM30) to understand the characteristics of mid-stance and double stance, as the max lateral displacement affecting balance typically occurs at mid-stance around 30% of the gait cycle [26]. The double stance accounts for about 10% of the total gait cycle after initial contact in a normal walking [27]. For older adults, since the double stance time was 15–20% of the entire gait cycle [28], we estimated the double stance as ±10% from the heel-strike and used such acceleration data. Table 2 provides a list of the extracted features with a description. The features were averaged from all steps (expressed by lowercase “a” at the beginning of each feature), and symmetry (expressed by lowercase “s” at the beginning of each feature) and variability (expressed by lowercase “v” at the beginning of each feature) were calculated for each stride (e.g., aM, sM, vM). In total, 60 features for each of the foot and pelvis sensors and 120 features for a combination of both foot and pelvis sensors were extracted for this study.

The extracted gait features from the lower limb and pelvis are subject to different magnitudes based on the individual features. To minimize the effects of individual feature differences, we normalized inertial gait features by using mean centering. Features were rescaled to have a zero mean by subtracting each feature’s mean from all observations on that feature in the dataset.

### 2.4. Data Analysis

#### 2.4.1. Feature Reduction Using Elastic Net

We aimed to build a linear regression model with feature selection that can predict TUG test scores. The two well-known penalized regression methods are the ridge regression [29] and least absolute shrinkage and selection operator (LASSO) [30] methods. While the ridge regression is better at adjusting for multi-collinearity among correlated features but cannot produce a sparse model, the LASSO tends to produce a sparser model by shrinking the coefficient of the less important variables to zero. To overcome the limitations of ridge regression and LASSO, as well as considering the strong points of both, the elastic net—a combination of ridge regression and LASSO—was proposed by Zou and Hastie [31]. The elastic net [31] showed a better performance for variable selection compared to the LASSO when the number of predictors (*p*) was much greater than the number of observations (*n*) in the presence of the multi-collinearity issue among predictors.

In this paper, therefore, we used the elastic net for a feature reduction method and this can be achieved by solving the following equation:

(1) $$\min_{\beta }\left[\frac{1}{n}\overset{n}{\underset{i=1}{\sum }}{\left(y_{i}-x_{l}^{T}\beta \right)}^{2}+\lambda \left(\left(1-\alpha \right)\frac{{||\beta ||}_{2}^{2}}{2}+\alpha {||\beta ||}_{1}\right)\right]$$

where *y_i_* and $x_{i}^{T}=\left(x_{i1},\ldots ,x_{ip}\right)$ are the respective outcome and predictors of the *i*th subject; λ is a non-negative tuning parameter, $\beta ={\left(\beta_{1},\ldots ,\beta_{p}\right)}^{T}$ is a vector of regression coefficients that needs to be estimated; and ${||\beta ||}_{1}$ and ${||\beta ||}_{2}$ are the regularization terms called *L*_1_-*norm* and *L*_2_-*norm*, respectively:

(2) $${||\beta ||}_{1}=\overset{p}{\underset{j=1}{\sum }}\left|\beta_{j}\right|$$

(3) $${||\beta ||}_{2}=\sqrt{\overset{p}{\underset{j=1}{\sum }}\beta_{j}^{2}}$$

Here, α represents the elastic net mixing parameter, which lies between 0 and 1 with α = 0 giving ridge regression and α = 1 giving LASSO. The model was run using alpha equals 0 to alpha equals 1 with a step of 0.1. We chose the best α using cross-validation (CV). The optimal penalty tuning parameter of the elastic net λ was chosen to minimize the mean square error (MSE) based on 10-fold CV. This was repeated 100 times for each penalty value with training data selected randomly from 70% of the data. The average prediction errors for test data were computed with the remaining 30% of the data. Based on the 100 iterations, we derived the top 10 most selected features from each sensor location (i.e., foot, pelvis, and a combination of both).

#### 2.4.2. Feature Selection and Model Fitting via Ridge Regression

Ridge regression shrinks the coefficients towards zero by minimizing the MSE of the estimates [29]. This is a regularization method used to analyze all data that have a multi-collinearity problem [15]. Since some of the top 10 features were highly correlated as shown in Table 3 and Table 4, the ridge regression was applied to build the best model to avoid multi-collinearity in linear regression.

The equation of the ridge regression estimator is:

(4) $${\hat{\beta }}_{ridge}={\left(X’X+kI\right)}^{-1}X’Y$$

where *X* is a design matrix for the predictors, Y is the response variable, *k* is a ridge tuning parameter, and *I* is an identity matrix. The ridge regression depends on the parameter *k,* which can affect the performance of the model and give information about the regularization [32]. If the parameter *k* is zero, then the ridge regression is restricted to the ordinary least squares (OLS) method [29]. Many studies investigated a method for calculating the optimal value of *k*. In this study, with *k* increasing by 0.001 between 0 and 2, the optimal parameter *k* was chosen in which the generalized CV (GCV) was the smallest as proposed by Golub et al. [33]. To determine the best model among the top 10 features, criteria that allow model comparison are essential. In this study, we used the Akaike Information Criterion (AIC) [34] and Bayesian Information Criterion (BIC) [35], which have been widely used in model comparison and model selection. In addition, the corrected AIC (AICc) [36] that has a correction for small sample sizes was used as another criterion for model selection. AIC, AICc, and BIC are defined as:

(5) $$\mathrm{AIC}=-2\ln L\left(\overset{\wedge }{\theta }\right)+2p$$

(6) $$\mathrm{AICc}=\mathrm{AIC}+\frac{2p^{2}+2p}{n-p-1}$$

(7) $$\mathrm{BIC}=-2\ln L\left(\overset{\wedge }{\theta }\right)+p\ln(n)$$

where 

$L(\overset{\wedge }{\theta })$ the likelihood of the model evaluated at the maximum likelihood estimate (MLE), *p* is the total number of parameters, and *n* is the number of observations. Lower AIC and BIC values indicate a better model fit. Thus, we compared the AIC and BIC values of the ridge regression models by adding features one-by-one among the top 10 features. The model that corresponded to the lowest AIC and BIC was referred to as “best” among the candidate models.

#### 2.4.3. Performance Evaluation

To compare the predictive accuracy for our best models constructed by using the different sensor locations, the following performance measures were calculated for the test data for each model:

- Mean square prediction error (MSPE)

(8) $$\mathrm{MSPE}=\frac{1}{n}\overset{n}{\underset{i=1}{\sum }}{\left[Tp\left(i\right)-Tm\left(i\right)\right]}^{2}$$

- Root mean square prediction error (RMSPE)

(9) $$\mathrm{RMSPE}=\sqrt{\frac{1}{n}\overset{n}{\underset{i=1}{\sum }}{\left[Tp\left(i\right)-Tm\left(i\right)\right]}^{2}}$$

- Mean absolute prediction error (MAPE)

(10) $$\mathrm{MAPE}=\frac{1}{n}\overset{n}{\underset{i=1}{\sum }}\left|Tp\left(i\right)-Tm\left(i\right)\right|$$

where *Tp(i)* and *Tm(i*) are the respective predicted and measured TUG (second) of the *i*th subject.

The performance of our model was evaluated using the following criteria. First, we split the whole data set (thirty-seven subjects) into a ratio of 7 to 3 such that 25 subjects were used for training and 12 subjects were used for testing. The penalized regression coefficients were determined by the TUG test scores for the training set. These coefficients were then used to predict the TUG test scores for subjects in the test set. This process was repeated 100 times using a random selection of training and test subjects for each iteration. In all comparisons, each model for the different sensors was executed using the same set of random selections, ensuring that the validation dataset was the same across models.

#### 2.4.4. Statistical Analysis and Software

For each prediction error, the predicted TUG test scores in the different models were compared using analysis of variance (ANOVA) with Tukey’s post hoc test for multiple comparisons at a 95% confidence level (if a *p*-value smaller than 0.05 was considered statistically significant). Bland–Altman analysis [37] was used to compare the estimated TUG and the measured TUG. Bland–Altman plots allow comparisons between two different measurements [37]. All analyses were performed using R statistical software (version 4.0.2). The *glmnet* package [38] was used to perform elastic net for feature selection. The ridge regression for model fitting was performed using the *lmridge* package [39].

## 3. Results

### 3.1. Elastic Net Results

**Figure 4:**
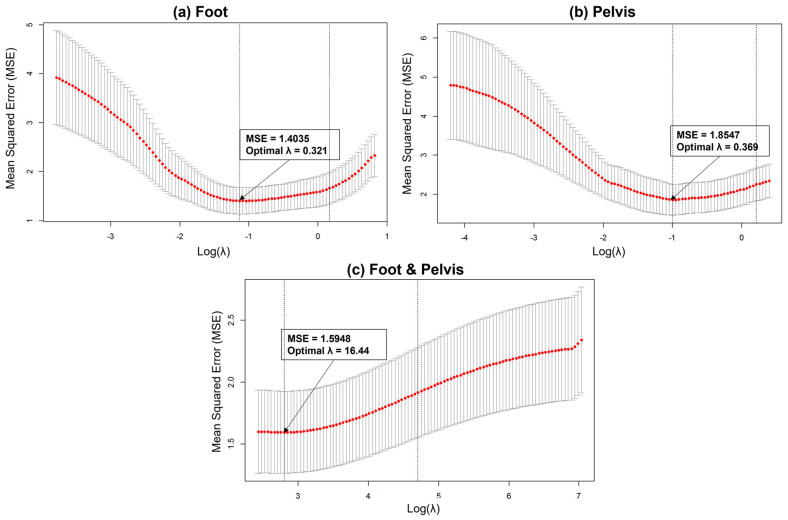
Optimal penalty parameter λ tuning for elastic net: (**a**) foot, (**b**) pelvis, and (**c**) foot and pelvis. The optimal λ for each model was chosen when the MSE was minimum (λ = 0.321, 0.369, and 16.44 for foot, pelvis, and foot & pelvis, respectively).

The optimal λ over the imputed data sets varied between 0.188 and 14.553 for the foot model, between 0.064 and 0.166 for the pelvis model, and between 0.2 and 33.674 for the combination model. Figure 4 illustrates the performance of all 100 penalty values, an example of the parameter tuning procedure in the elastic net. The averaged optimal λ values were 4.821, 4.037, and 8.401 for the foot, pelvis, and combination models, respectively.

Based on these results, the top 10 most selected features were derived for each model across the imputed data sets. As there existed a tie in the number of selected features for the foot–pelvis combination model, we included the top 11 features for the foot–pelvis combination model. The feature ‘aM’ was the most selected in both foot and pelvis models (Table 5). The whole-step-related (aM, sM, vM, vST, sST, aVM, and vAM) and double-stance-related (aVMD, aMD, and aAMD) features were mostly selected for the foot model. The initial 10% of step-related features (aVHS, aAHS, aM10, sVHS, aVHM, and sM10) were mostly selected for the pelvis model. The mid-stance-related feature (sM30) was selected only for the pelvis model. The top 10 features selected for the foot–pelvis combination model included features selected from both models. This result shows that the top 10 features were consistently selected in different models.

### 3.2. Feature Selection and Model Fitting

**Figure 5:**
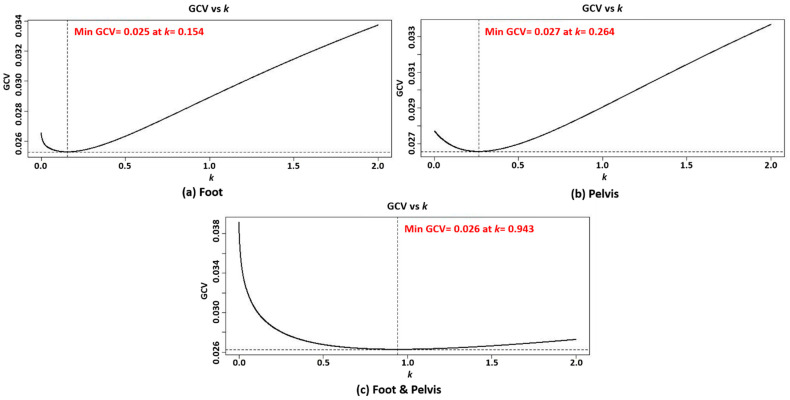
Optimal parameter *k* tuning for ridge regression: (**a**) foot, (**b**) pelvis, and (**c**) foot and pelvis. The optimal *k* for each model was chosen when the GCV was minimum (*k* = 0.154, 0.264, and 0.943 for foot, pelvis, and foot & pelvis, respectively).

For ridge regression, the optimal parameter *k* was selected based on the minimum value of GCV [33]. Figure 5 illustrates the procedure for finding the parameter *k.* The results for all models with the top 10 features are summarized in Table 5. In order to choose the best model, we also calculated the AIC, AICc, and BIC values for each model that are also summarized in Table 6, and then the values were compared. The best models were selected with the smallest AIC and BIC values as follows: top five features for the foot model (AIC: −2.845, BIC: 136.377), top four features for the pelvis model (AIC: −0.864, BIC: 137.327), and top 10 features for the combination model (AIC: −1.377, BIC: 137.510).

### 3.3. Prediction and Validation Results

**Figure 6:**
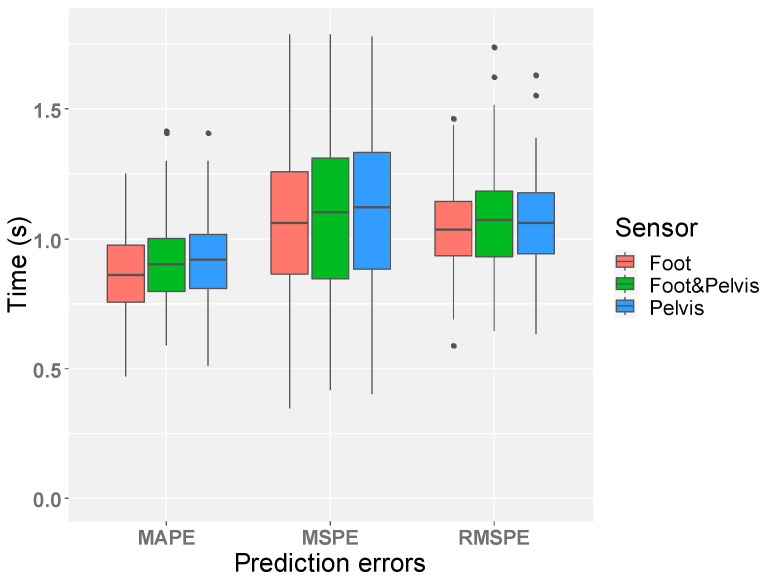
Result of prediction errors. The distribution of each prediction error for the 100-time iterations is shown (The mean values for MAPE are foot (0.865 s), pelvis (0.921 s), and foot & pelvis (0.918 s), for MSPE are: foot (1.124 s), pelvis (1.162 s), and foot & pelvis (1.192 s), and for RMSPE are foot (1.046 s), pelvis (1.065 s), and foot & pelvis (1.075 s)). There was no significant difference of prediction errors between different sensor locations.

**Figure 7:**
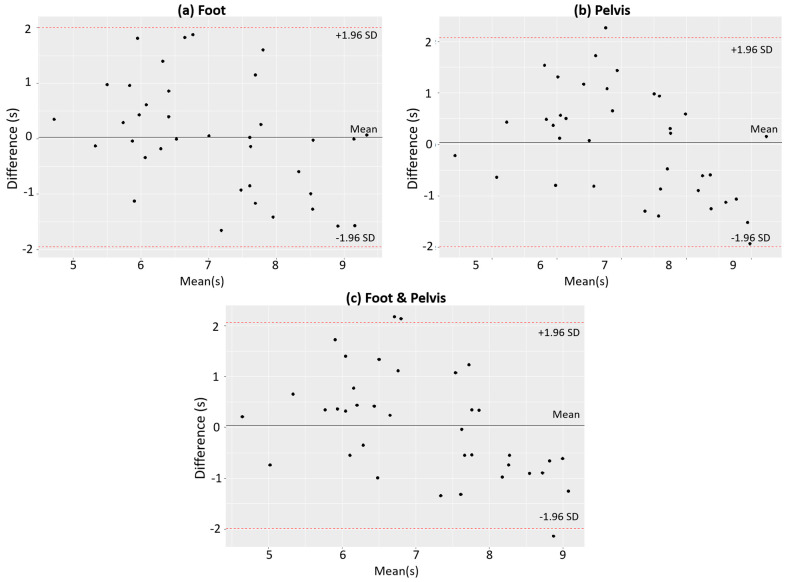
Bland–Altman plot comparing predicted TUG and actual TUG: (**a**) foot, (**b**) pelvis, and (**c**) foot and pelvis. The 95% limits of agreement are shown as two red dotted lines.

The prediction errors for the selected best models are shown in Figure 6. For comparison of the best models among different sensors, the foot model performed better than others (MAPE: foot (0.865 s) > foot & pelvis (0.918 s) > pelvis (0.921 s), MSPE: foot (1.124 s) > pelvis (1.162 s) > foot & pelvis (1.192 s), and RMSPE: foot (1.046 s) > pelvis (1.065 s) > foot & pelvis (1.075 s)). The result of multiple comparisons between different sensor models showed that there was no significant difference of prediction errors between them. The *p*-values for all cases were greater than 0.05 as shown in Table 7. A comparison between the estimated TUG and the measured TUG by Bland–Altman analysis [37] is shown in Figure 7. In addition, the best models were compared with other models that used different numbers of features to see if the best models gave the best prediction results. The comparison results summarized in Table 8 showed that the best foot model performed optimally for all performance criteria, while the best pelvis model performed optimally for MSPE and RMSPE but not for MAPE, and the performance of the best foot–pelvis combination model was not optimal for all criteria.

## 4. Discussion

The results of this study show that TUG scores can be predicted through walking data of older adults obtained using wearable sensors. A set of the best gait features and a ridge regression model were used to develop a TUG prediction model. To check the reliability of the developed model, we used Bland–Altman analysis, which is a simple and accurate way to compare different measurements [37]. In Figure 7, the mean difference for all three plots was on the order of 1 s. This indicates that two different measurements are not systematically producing different results. In addition, we also compared our results with Saporito’s work. The 95% limits of agreement in the Bland–Altman plots were narrower than those of Saporito’s Bland–Altman plots, which means our estimated TUG is essentially equivalent to the measured actual TUG. Saporito and colleagues developed the remote TUG prediction model with 2.1 s of MAPE [23]. Thus, the developed model demonstrated prediction errors (foot: MSPE = 1.124s, RMSPE = 1.046 s, MAPE = 0.865 s, pelvis: MSPE = 1.162 s, RMSPE = 1.065 s, MAPE = 0.921 s, foot–pelvis combination: MSPE = 1.192 s, RMSPE = 1.075 s, MAPE = 0.918 s) that are less than Saporito’s prediction errors. We also described the advantages and disadvantages of the proposed method compared to Saporito’s method as shown in Table 9. Furthermore, our results show that the developed model can be an objective tool used clinically. By predicting TUG scores, this model can objectively identify changes in function related to fall risk in older adults [40,41,42].

This study also determined the optimal IMU sensor locations and a set of features for predicting the TUG scores. Our results determined that IMU sensors located on the feet had the better TUG score prediction. The increased accuracy of the model using the foot sensor location could be due to the sensor being closer to the ground and therefore experiencing more changes in acceleration throughout the gait cycle for each step. Nevertheless, the location of the pelvis sensor is less obtrusive than that of the foot sensor, as the pelvis sensor can be attached to the waist belt. From a practical point of view, the single pelvis sensor’s prediction error has no significant difference compared to the foot sensor (Table 7) and it has less interference in real-life. Moreover, from a clinical point of view, many researchers have already verified the use of a single pelvis sensor for evaluating the spatio–temporal parameters of walking in healthy subjects and in patients [43,44,45]. Since spatial and temporal parameters are commonly used as major indicators for characterizing gait [46] and can provide clinically meaningful information related to the patient’s state and the progression of certain diseases [47], the single pelvis sensor will offer the clinicians not only the ability to predict TUG but also to obtain data related to spatio-temporal parameters. Therefore, it can be concluded that the single pelvis sensor is recommended for monitoring the risk of falls in older adults by TUG estimation. The results also determined that the best set of features for predicting the TUG score using the foot sensor was aM, sM, vM, aVMD, and aMD. This feature set provides insight into the characteristics of each step in the data. The average (aM) whole step vector magnitude characterizes the accelerations of the foot sensor throughout each whole step, while the variability (vM) characterizes how much the accelerations change for each whole step. The symmetry (sM) of the whole step vector magnitude characterizes how similar the left and right foot sensor accelerations are. These features may have been selected by the model as they provide an overall insight into how a participant walks. The average vector magnitude (aMD) and average vertical vector magnitude (aVMD) during double stance features were also selected. The double stance phase in the gait cycle is also a transition phase when body weight moves from the stance leg to the swing leg [48,49,50,51]. The model may have chosen aMD and aVMD as features, as double stance is a phase in which falls are likely to occur due to changes in body weight from one leg to the other [48,49,50,51]. For the pelvis sensor, the best set of features was aM, aMD, vST, and sM30. Mostly similar features were selected, but the variability of the step time (vST) was also selected, which can be an obviously effective predictor of TUG. It is expected that the feature sets will have the potential to be used sufficiently to predict not only TUG but also other balance evaluation tests.

This study has several limitations. The first limitation is that the setup for the walking task was not performed in a daily-life setting, which could make subjects’ walking artificial. The data obtained from a daily-life setting will be the closest to the most natural walking movement. However, it is not easy to accurately classify walking data without a separate monitoring device in a typical daily-life setting. Multiple monitoring devices also reduce the efficiency of data analysis by mass-producing worthless data. Our aim is to predict the TUG test score by measuring their gait with wearable devices. Therefore, we selected sidewalk walking that can occur frequently in daily-life and tried to make it as natural as possible when collecting data. The second limitation is that the data set is small and is limited to healthy older adults, making it difficult to draw general conclusions about the results. However, to overcome this limitation, the study repeatedly drew conclusions by randomly selecting training data and using independent validation sets. In addition, since this study is an early stage of research to predict the clinical assessments of older adults, it can serve as a steppingstone for building objective indicators to judge the health of older adults in the future. Another potential limitation of the dataset is that the smallest AIC and BIC were produced using 11 features for the foot–pelvis combination model, as the number of observations was not enough compared to the number of predictors. This can cause the danger of overfitting the model. In Table 6, our results showed that the foot–pelvis combination model built with the smallest AICc performed better than the one built with the smallest AIC and BIC. For future studies, a larger sample size should be used to support the level of complexity. The other limitation of this study is that participants are relatively healthy older adults. Experiments with more diverse groups of older adults would have produced more discriminative results in predicting TUG scores as well as the risk of falling. Our future research plan is to use more data sets that include patients with balance issues to investigate the power of our model in predicting various clinical assessments and mitigating the impact of smaller datasets and to improve predictive models for remotely monitoring older adults’ mobility by collecting data from actual daily-life settings.

## 5. Conclusions

This study was conducted to determine if TUG scores, a typical balance assessment, could be predicted in older adult gait in a community-dwelling environment, not in a laboratory setting. The proposed prediction model using the ridge regression in this study showed satisfactory results in predicting the TUG scores. We also conducted experiments to find out in which position the sensor should be worn to help predict the TUG scores. The results determined that the sensor attached to the pelvis was recommended for predicting the TUG scores of older adults due to the simplicity of a single sensor and less obstruction compared to two feet or foot–pelvis combination sensors, as well as for obtaining data related to spatio–temporal parameters for clinically meaningful information. Through this study, we not only developed a model that reliably predicts the TUG scores of older adults, but also left open the possibility of developing a health monitoring system through the daily walking of older adults by being able to predict the risk of falls in older adults.

## Figures and Tables

**Table 1:** Descriptive characteristics of participants.

Characteristics	Mean ± Standard Deviation
Number of participants	37
Female/male	29/8
Age (years)	69.6 ± 4.3
Height (cm)	166.1 ± 11.2
Weight (kg)	75.1 ± 15.0
BMI (kg/m^2^)	27.1 ± 4.1
TUG (s)	7.1 ± 1.5

**Table 2:** Description of extracted features.

Feature	Description	Mathematical Expression
M	Whole step vector magnitude	$\sqrt{L^{2}+V^{2}+AP^{2}}$ for whole step vectors
M10	Initial 10% step vector magnitude	$\sqrt{L^{2}+V^{2}+AP^{2}}$ for initial 10% of step vectors
LM	Lateral vector magnitude during a whole step	$\sqrt{L^{2}}$ for whole step vectors
VM	Vertical vector magnitude during a whole step	$\sqrt{V^{2}}$ for whole step vectors
AM	AP vector magnitude during a whole step	$\sqrt{AP^{2}}$ for whole step vectors
MD	Vector magnitude during double stance	$\sqrt{L^{2}+V^{2}+AP^{2}}$ for ± 10% vectors from heel-strike
LMD	Lateral vector magnitude during double stance	$\sqrt{L^{2}}$ for ± 10% vectors from heel-strike
VMD	Vertical vector magnitude during double stance	$\sqrt{V^{2}}$ for ± 10% vectors from heel-strike
AMD	AP vector magnitude during double stance	$\sqrt{AP^{2}}$ for ± 10% vectors from heel-strike
M30	Vector magnitude during mid-stance	$\sqrt{L^{2}+V^{2}+AP^{2}}$ for vectors from 30% of gait cycle
LM30	Lateral vector magnitude during mid-stance	$\sqrt{L^{2}}$ for vectors from 30% of gait cycle
VM30	Vertical vector magnitude during mid-stance	$\sqrt{V^{2}}$ for vectors from 30% of gait cycle
AM30	AP vector magnitude during mid-stance	$\sqrt{AP^{2}}$ for vectors from 30% of gait cycle
LHM	Lateral heel-strike magnitude	max(L) at heel-strike
LHS	Std. of lateral acceleration during initial 10% step	std(L) for initial 10% of step vectors
VHM	Vertical heel-strike magnitude	max(V) at heel-strike
VHS	Std. of vertical acceleration during initial 10% step	std(V) for initial 10% of step vectors
AHM	AP heel-strike magnitude	max(AP) at heel-strike
AHS	Std. of AP acceleration during initial 10% step	std(AP) for initial 10% of step vectors
ST	Step Time	Time between opposite heel strikes

Std.: Standard Deviation, L: lateral acceleration, V: vertical acceleration, AP: anterior-posterior acceleration.

**Table 3:** Correlation matrix of the top 10 features for the foot (* *p*-value < 0.05).

Feature	aM	aVM	aMD	aVMD	aAMD	sM	sST	vM	vAM	vST
**aM**	1.000									
**aVM**	0.667 *	1.000								
**aMD**	0.690 *	0.289	1.000							
**aVMD**	0.617 *	0.303	0.975 *	1.000						
**aAMD**	0.732 *	0.195	0.727 *	0.622 *	1.000					
**sM**	−0.099	−0.139	0.169	0.197	0.069	1.000				
**sST**	0.202	0.303	0.188	0.147	0.221	0.157	1.000			
**vM**	0.088	0.005	0.168	0.205	0.068	−0.096	−0.208	1.000		
**vAM**	0.085	0.004	0.117	0.127	0.071	−0.129	−0.121	0.930 *	1.000	
**vST**	−0.134	−0.167	−0.052	0.006	−0.119	0.046	−0.356 *	0.841 *	0.816 *	1.000

**Table 4:** Correlation matrix of the top 10 features for the pelvis (* *p*-value < 0.05).

Feature	aM	aM10	aMD	aVHM	aVHS	aAHS	sM10	sM30	sVHS	vST
**aM**	1.000									
**aM10**	0.456 *	1.000								
**aMD**	0.411 *	0.917 *	1.000							
**aVHM**	0.253	0.817 *	0.809 *	1.000						
**aVHS**	0.678 *	0.697 *	0.712 *	0.646 *	1.000					
**aAHS**	0.838 *	0.246	0.177	−0.079	0.515 *	1.000				
**sM10**	−0.317	−0.333 *	−0.539 *	−0.314	−0.463 *	−0.216	1.000			
**sM30**	−0.508 *	−0.582 *	−0.690 *	−0.399 *	−0.709 *	−0.407 *	0.722 *	1.000		
**sVHS**	0.000	−0.267	−0.196	−0.294	−0.023	0.133	0.025	0.025	1.000	
**vST**	−0.365 *	−0.087	−0.054	−0.030	−0.150	−0.382 *	−0.163	−0.037	−0.083	1.000

**Table 5:** Top 10 most selected features.

Rank	Foot		Pelvis		Foot & Pelvis	
	Feature	Frequency	Feature	Frequency	Feature	Frequency
1	aM	100	aM	98	aMD_Foot	98
2	aVMD	97	aMD	97	aVMD_Foot	98
3	aMD	86	vST	97	aVHS_Pelvis	98
4	sM	78	sM30	91	aM_Pelvis	97
5	vM	78	aVHS	82	aM_Foot	93
6	vST	76	aAHS	79	aMD_Pelvis	93
7	sST	72	aM10	77	sM30_Pelvis	93
8	aAMD	67	sVHS	70	vST_Pelvis	93
9	aVM	65	aVHM	69	aM10_Foot	89
10	vAM	64	sM10	65	aAHS_Pelvis	88
	-	-	-	-	vST_Foot	88

The column “Frequency” gives the number of times each feature was selected.

**Table 6:** AIC/AICc/BIC Values for Model Selection.

No. of Features	Foot				Pelvis				Foot & Pelvis			
	*k*	AIC	AICc	BIC	*k*	AIC	AICc	BIC	*k*	AIC	AICc	BIC
2	0.174	6.882	7.235	143.05	0.105	3.468	3.821	139.94	0.166	11.163	11.516	146.46
3	0.275	7.161	7.888	143.35	0.147	−0.090	0.637	137.61	0.253	6.865	7.592	143.14
4	0.204	4.683	5.933	142.31	0.264	−**0.864**	**0.386**	**137.33**	0.379	3.068	4.318	139.97
5	0.154	−**2.845**	−**0.910**	**136.38**	0.366	0.129	2.064	138.56	0.560	3.745	5.680	140.69
6	0.236	−1.897	0.903	137.55	0.440	0.752	3.552	139.46	0.677	3.705	6.505	140.73
7	0.289	−1.722	2.140	138.60	0.560	1.282	5.144	139.96	0.859	3.929	7.791	141.17
8	0.345	−0.372	4.771	140.42	0.523	0.117	5.260	139.92	0.628	-0.938	**4.205**	137.71
9	0.430	−0.061	6.606	140.61	0.498	−0.762	5.905	139.56	0.681	−0.529	6.138	138.20
10	0.599	0.333	8.795	140.91	0.568	−0.850	7.612	139.83	0.806	−0.449	8.013	138.34
11	-	-	-	-	-	-	-	-	0.943	−**1.377**	9.183	**137.51**

The name of the selected features for each model is shown in Table 5.

**Table 7:** Multiple comparisons between different models.

Error	Model 1	Model 2	*p*-Value
**MAPE**	Foot	Pelvis	0.0507
	Foot	Foot & Pelvis	0.0655
	Pelvis	Foot & Pelvis	0.9942
**MSPE**	Foot	Pelvis	0.7674
	Foot	Foot & Pelvis	0.4444
	Pelvis	Foot & Pelvis	0.8608
**RMSPE**	Foot	Pelvis	0.7389
	Foot	Foot & Pelvis	0.4925
	Pelvis	Foot & Pelvis	0.9181

**Table 8:** Comparison of Prediction Errors.

**No. of Features**	**MSPE**			**RMSPE**			**MAPE**		
	**Foot**	**Pelvis**	**Foot & Pelvis**	**Foot**	**Pelvis**	**Foot & Pelvis**	**Foot**	**Pelvis**	**Foot & Pelvis**
2	1.386	1.231	1.536	1.165	1.100	1.225	0.954	0.942	1.040
3	1.418	1.180	1.370	1.178	1.073	1.158	0.962	0.931	0.985
4	1.313	**1.162**	1.232	1.134	**1.065**	1.100	0.941	**0.921**	0.926
5	**1.124**	1.187	1.255	**1.046**	1.078	1.109	**0.865**	0.932	0.924
6	1.264	1.218	1.258	1.108	1.091	1.110	0.917	0.932	0.931
7	1.255	1.226	1.262	1.103	1.095	1.112	0.927	0.935	0.933
8	1.309	1.217	1.173	1.126	1.089	1.068	0.951	0.910	0.909
9	1.305	1.232	1.168	1.124	1.094	1.068	0.954	0.915	0.910
10	1.267	1.306	1.168	1.109	1.115	1.067	0.936	0.938	0.905
11	-	-	**1.192**	-	-	**1.075**	-	-	**0.918**

The name of selected features for each model is shown in Table 5.

**Table 9:** Comparison of the advantages and disadvantages of our study with those of Saporito.

Advantages	Disadvantages
It is difficult to compare the prediction errors directly because the methods used between the two studies are different, but the TUG prediction error of our study was lower than Saporito’s prediction error. Our study estimated the TUG test score using three minutes of walking data, whereas Saporito’s needed three days of activities of daily living. This proves that 3-min walking data are enough to predict the TUG test.	Although both studies reasonably estimated the TUG test in older adults, the subjects of our study were relatively healthy. Saporito had a wider range of TUG values. Our study conducted the experiment as close to a real-life setting as possible, but it was still semi-realistic. On the other hand, Saporito experimented in a free-living environment.
